# Identification of a Metabolism-Related Risk Signature Associated With Clinical Prognosis in Glioblastoma Using Integrated Bioinformatic Analysis

**DOI:** 10.3389/fonc.2020.01631

**Published:** 2020-09-03

**Authors:** Zheng He, Chengcheng Wang, Hao Xue, Rongrong Zhao, Gang Li

**Affiliations:** ^1^Department of Neurosurgery, Qilu Hospital, Cheeloo College of Medicine, Shandong University, Jinan, China; ^2^Shandong Key Laboratory of Brain Function Remodeling, Jinan, China; ^3^Institute of Brain and Brain-Inspired Science, Shandong University, Jinan, China; ^4^Department of Pharmacy, Qilu Hospital (Qingdao), Cheeloo College of Medicine, Shandong University, Qingdao, China

**Keywords:** glioblastoma, metabolism, prognosis, signature, heterogeneity

## Abstract

Altered metabolism of glucose, lipid and glutamine is a prominent hallmark of cancer cells. Currently, cell heterogeneity is believed to be the main cause of poor prognosis of glioblastoma (GBM) and is closely related to relapse caused by therapy resistance. However, the comprehensive model of genes related to glucose-, lipid- and glutamine-metabolism associated with the prognosis of GBM remains unclear, and the metabolic heterogeneity of GBM still needs to be further explored. Based on the expression profiles of 1,395 metabolism-related genes in three datasets of TCGA/CGGA/GSE, consistent cluster analysis revealed that GBM had three different metabolic status and prognostic clusters. Combining univariate Cox regression analysis and LASSO-penalized Cox regression machine learning methods, we identified a 17-metabolism-related genes risk signature associated with GBM prognosis. Kaplan-Meier analysis found that obtained signature could differentiate the prognosis of high- and low-risk patients in three datasets. Moreover, the multivariate Cox regression analysis and receiver operating characteristic curves indicated that the signature was an independent prognostic factor for GBM and had a strong predictive power. The above results were further validated in the CGGA and GSE13041 datasets, and consistent results were obtained. Gene set enrichment analysis (GSEA) suggested glycolysis gluconeogenesis and oxidative phosphorylation were significantly enriched in high- and low-risk GBM. Lastly Connectivity Map screened 54 potential compounds specific to different subgroups of GBM patients. Our study identified a novel metabolism-related gene signature, in addition the existence of three different metabolic status and two opposite biological processes in GBM were recognized, which revealed the metabolic heterogeneity of GBM. Robust metabolic subtypes and powerful risk prognostic models contributed a new perspective to the metabolic exploration of GBM.

## Introduction

Glioblastoma (GBM) is the most common, most aggressive and worst prognosis glioma in adults (1), accounting for about 55% of gliomas, with median survival of only 14–16 months (1, 2). The diffusivity and invasiveness of GBM itself and the inter-/intra-tumor heterogeneity lead to GBM therapy resistance and high recurrence (3–6). Therefore, despite the standard treatment protocol for GBM such as surgical resection, radiotherapy and chemotherapy, the prognosis of GBM still remains dismal, and the 5-year survival rate is only about 5% (2, 7, 8). Novel molecular markers or therapeutic targets are urgently needed to improve the prognosis of GBM.

Metabolic reprogramming is one of the hallmarks of cancer cells, and there is growing evidence that metabolic dysregulation plays an important role in the growth, proliferation, angiogenesis, and invasion of cancer cells (9–11). Civita et al. (12) revealed the landscape of GBM heterogeneity using laser capture microdissection and RNA-seq analysis, which showed dysregulation of metabolic pathways, providing direct evidence for metabolic alterations in GBM. The metabolism of glucose, lipid, and glutamine in cancer cells is altered (13). According to Warburg's basic research, cancer cells obtain energy mainly through the glycolytic pathway rather than the oxidative phosphorylation (OxPhos) pathway, so abnormal glycolytic metabolism is one of the basic characteristics of malignant cells (14). Recent studies have found that lipid metabolism reprogramming plays a crucial role in membrane synthesis, energetic production and signal transduction in the progression of cancer cells (15). Nuclear magnetic resonance (NMR) spectroscopy revealed that unsaturated fatty acids, cholesterol esters and phosphatidylcholine are only present in the GBM (16, 17). At present, the biological phenotype and molecular mechanism of lipid composition change leading to glioma need further study. In addition, studies on cancer cell metabolism have provided evidence that tumor-specific activation of signaling pathways, such as the upregulation of the oncogene *Myc*, can regulate glutamine uptake and its metabolism through glutaminolysis to provide the cancer cell with a replacement of energy source (18). Therefore, the in-depth exploration of three major metabolites of GBM may provide an important theoretical basis for the development of new treatment (10, 19). However, there has been no comprehensive analysis of the three-major metabolism-related genes and their prognostic value in GBM.

In the present study, we comprehensively analyzed GBM mRNA sequencing data from three public datasets, the Cancer Genome Atlas (TCGA), The Chinese Glioma Genome Atlas (CGGA), and GSE13041, to explore the metabolic status of GBM patients. Through cluster analysis, the patients can be divided into 3 stable clusters according to the gene expression profile, and the prognosis and molecular characteristics of the 3 clusters are significantly different. In addition, we further screened potential specific therapeutic compounds for each cluster. More importantly, we identified a metabolism-related risk signature to assess the prognosis of patients with GBM in the TCGA datasets which can be served as an independent predictor closely related to the prognosis of GBM patients. And the high- and low-risk groups had distinctly different biological processes. The above results were further validated in CGGA and GSE13041 datasets. In summary, robust prognostic risk models and subtypes contribute to a better understanding of the molecular pathogenesis of GBM.

## Materials and Methods

### Data Collection

Whole genome mRNA expression sequencing data and corresponding clinical information [histology, subtype, gender, age, isocitrate dehydrogenase1 (IDH1) mutational status, methylguanine methyltransferase (MGMT) promoter status, glioma cytosine-phosphate-guanine island methylator phenotype (G-CIMP) status and survival information] of 165 GBM patients were downloaded from TCGA (PanCancer Atlas) (20) datasets as training set. Similarly, GSE13041 (21) and CGGA RNA expression data and clinical information were obtained as validation sets. Of which, mRNA sequencing data from CGGA contains two datasets, mRNAseq_693 and mRNAseq_325, whose platforms are Illumina HiSeq and Illumina HiSeq 2000 or 2500, respectively. Therefore, we removed the batch effect from these two datasets and normalized them to obtain an integrated data of 216 GBM patients. The characteristics of GBM patients from these three datasets were summarized in Supplementary Table 1. Five hundred and thirty two Glucose-, 1034 lipid-, and 13 glutamine-metabolism related genes were downloaded from Molecular Signature Database v7.0 (MSigDB) (http://www.broad.mit.edu/gsea/msigdb/) (22). The detailed metabolism-related genes were listed in the Supplementary Table 2.

### Consensus Clustering

We took the expression profile of metabolism-related genes for consistent clustering by using “Cancersubtype” R package (http://cran.r-project.org). The euclidean distance was applied to calculate the similarity distance between samples, and K-means methods was utilized for clustering. By performing resampling analysis, 80% of the samples were sampled for 100 times. The optimal number of clusters was determined by the cumulative distribution function (CDF) and was validated in the CGGA and GSE13041 datasets. And principal component analysis (PCA) was carried out using the R package “princomp” to validate the molecular subtype.

### Gene Signature Identification

Univariate Cox regression were performed on the 169 GBM patients in TCGA datasets to select the optimal prognostic gene set with R package “glmnet.” After getting the corresponding hazard ratio (HR) and *p*-value of each gene, the genes with *p* < 0.05 were selected as seed genes for Cox LASSO regression with 10-fold cross-validation (CV). Risk score for each patient of the TCGA training set was calculated with the linear combinational of the signature gene expression (expr) weighted by their regression coefficients (23).

$$\begin{array}{l} \text{Risk score}=\text{exp}{\text{r}}_{\text{gene}1}\times {\text{β}}_{\text{gene}1}+\text{exp}{\text{r}}_{\text{gene}2}\times {\text{β}}_{\text{gene}2}\\ \;+\ldots +\text{exp}{\text{r}}_{\text{geneN}}\times \;{\text{β}}_{\text{geneN}}. \end{array}$$

In the above equation, “N” was the total number of key genes, “expr” represented the expression value of geneN, and β represented the selected gene coefficient from LASSO analysis. Patients in the training datasets was then categorized into high and low risk score groups according to the median risk score. The risk score for each patient in the validation datasets were also calculated based on the same risk formula. The multivariate Cox regression analysis was conducted to determine whether the risk score was an independent predictor for GMB patients with R package “survival.” The differences in overall survival (OS) between high-risk and low-risk score in the training and validation datasets were estimated using the Kaplan-Meier (KM) method. Receiver operating characteristic (ROC) analysis was performed to evaluate the accuracy of the risk model with R package “pROC.”

### Gene Set Enrichment Analysis (GSEA)

GSEA was performed using Gene Set Enrichment Analysis v3 software downloaded from the Broad Institute (http://www.broadinstitute.org/gsea/index.jsp) (24). The mRNA expression profile of GBM samples from the TCGA/CGGA/GSE13041 datasets were analyzed by GSEA. For GSEA, risk score was selected as a binary variable divided into high- and low-risk by a criterion of whether the score was greater than the median value. The collection of annotated gene sets of c2.cp.kegg.v7.0.symbols.gmt in MSigDB was chosen as the reference gene sets in GSEA software, the NOM *p* < 0.05 and false discovery rate (FDR) <0.25 was set as the cutoff.

### Connectivity Map (CMap) Analysis

CMap is a systematic, data-driven program for detecting correlations among genes, compounds, and biological conditions. We queried the recently updated CMap to screen potential compounds that might target metabolism-related pathways. A list of differential expression genes (DEGs) among the 3 clusters in TCGA was obtained using the “lmFit” function of the R package “limma” with default parameters (25), and the top 373 genes (148 upregulated and 225 downregulated) were selected to uploaded into the CMap database. Compounds with an absolute value of enrichment ≥ 0.7 and *p* < 0.05 were selected as potential therapeutic drugs for GBM.

### Statistical Analysis

One-way ANOVA was performed to compare the differences of risk score between/among subtypes/clusters. The one-way ANOVA method and Tukey's test was applied to identify the DEGs between the high- and low-risk groups (*q* < 0.05, |log2FC| > 2). KM curve with log-rank test was used to assess the OS differences among/between different groups. Univariate and multivariate Cox regression analyses were conducted to assess the independent prognostic factors. The statistical analyses were conducted using R software version 3.5.1 (R Core Team, R Foundation for Statistical Computing, Vienna, Austria), *p* < 0.05 was regarded as statistically significant.

## Results

### Molecular Cluster Identification and Validation

**Figure 1:**
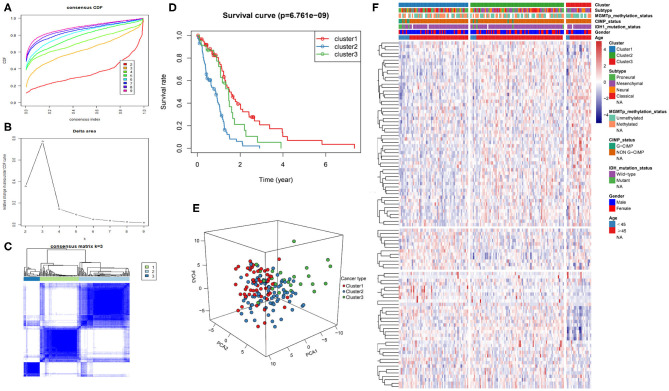
Metabolism-related genes could distinguish GBM patients in TCGA with different clinical and molecular features. **(A)** Consensus clustering CDF for k = 2 to k = 9. **(B)** Relative change in area under CDF curve for k = 2 to k = 9. **(C)** Consensus clustering matrix heatmap plots of 165 samples from TCGA datasets for k = 3. **(D)** Kaplan-Meier analysis of patients among 3 clusters. **(E)** PCA analysis of the metabolism-related genes expression when k = 3. **(F)** Heatmap of three clusters defined by the top 100 variable expression genes. CDF, cumulative distribution function; PCA, principal components analysis.

The intersection of the three datasets and metabolism genes was extracted, and the overlapping genes were removed. The gene expression profiles of 1,395 metabolism-related genes (Supplementary Table 2) were exploited to identify the GBM clusters in TCGA cohort. All GBM samples were grouped into k (k = 2, 3, 4, 5, 6, 7, 8, 9) different subtypes using “Cancersubtype” R package. According to the CDF curves of the consensus score, we selected the k = 3 as the optimal division (Figures 1A–C and Supplementary Figure 1). KM analysis showed that the OS of the 3 clusters were significantly different (Figure 1D, *p* < 0.05), and PCA revealed that the 3 clusters could be separated from each other (Figure 1E). Figure 1F showed the heatmap of these 3 clusters defined by the top 100 variable expression genes. The above results indicated that there were significant metabolic phenotypes among the 3 clusters. In order to validate the stability of molecular subtypes, we further selected CGGA and GSE13041 datasets for clustering. The clustering results of molecular subtypes in CGGA and GSE13041 datasets were consistent with those in TCGA, and the relevant results were shown in Supplementary Figures 2–5, respectively. Therefore, we identified three stable clusters of GBM based on the expression of metabolism-related genes.

### Identification of Metabolism-Related Genes Signature for Prognostic Prediction

**Figure 2:**
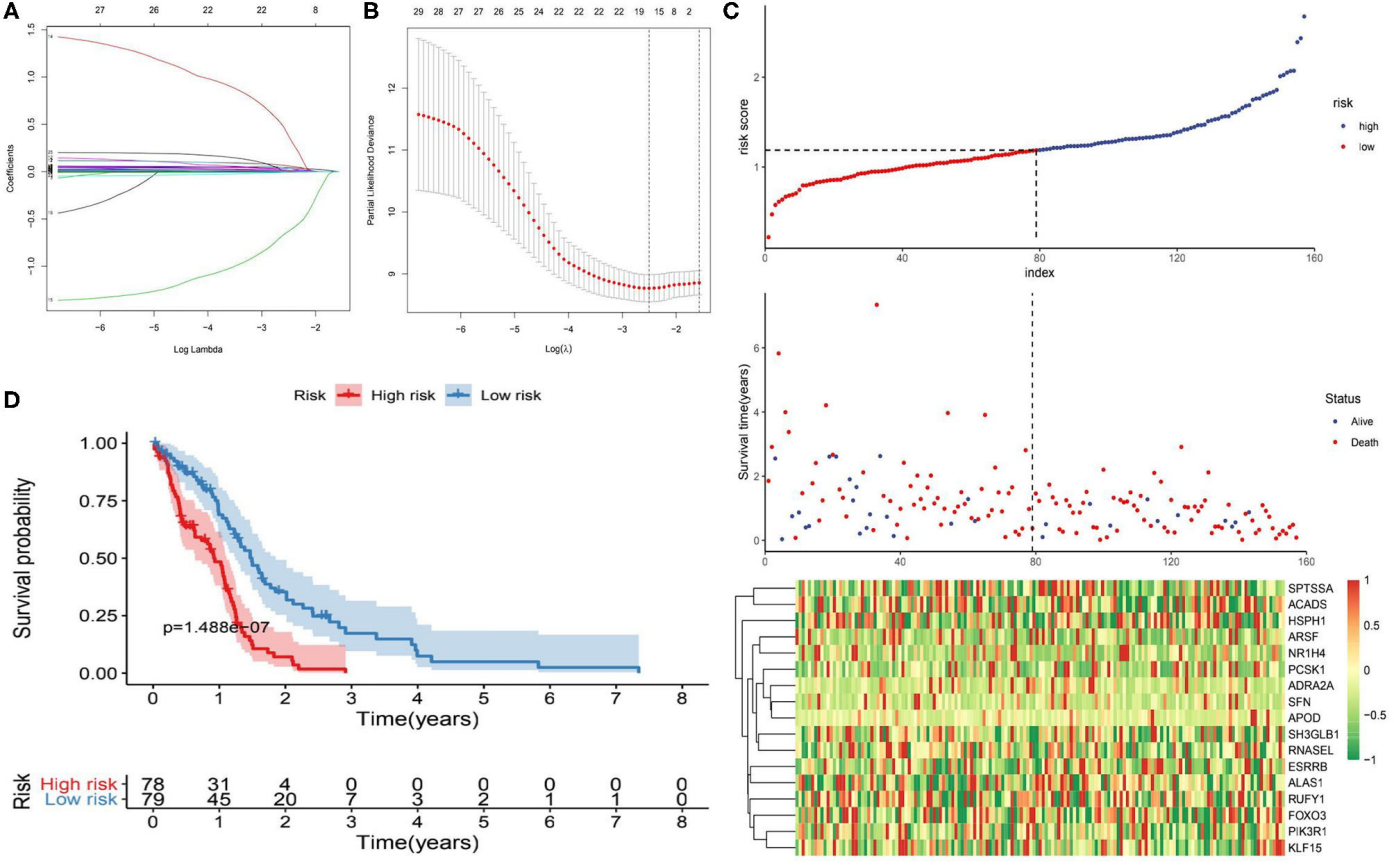
Identification of metabolism-related signature by Cox proportional hazards model in TCGA cohort. **(A)** LASSO coefficient profiles of the 29 survival-associated metabolism-related genes. **(B)** Cross-validation for tuning parameter selection in the proportional hazards model. **(C)** Risk plot for the GBM patients in TCGA datasets. Each panel consists of three rows: top row showed the risk score distribution for the high- and low-risk score group; middle row represents the GBM patients' distribution and survival status; the bottom row shows that the heatmap of 17 prognostic metabolism-related genes expression. **(D)** Kaplan-Meier curves analysis for the risk model in the TCGA datasets.

The 1,395 putative metabolism-related genes were exploited to conducting univariate cox regression analysis. Firstly, we identified 29 significantly metabolism-related genes associated with the survival of GBM with *p* < 0.05 (Supplementary Table 2). We further performed LASSO Cox regression algorithm with cross-validation (Figures 2A,B), after 1,000-time iterations, a 17-gene risk signature was constructed (Table 1) and the risk score for each patient was calculated with their expression level and regression coefficient. To comprehensively investigate the relationship between risk score and patients' survival, we further stratified patients into high- and low-risk groups based on the median risk score (Figure 2C). And as shown in Figure 2C, patients of GBM in TCGA cohort with high risk score have more death cases when compare to low risk score. KM curves analysis result revealed that patients in high risk group had a shorter survival time than in the low risk group (Figure 2D). To validate this gene set, we also calculated patients' risk scores of CGGA and GSE13041 cohorts with same regression coefficient. And as expected, consensus result was also obtained in the validation datasets (Supplementary Figure 6).

**Table 1:** The 17 metabolism-related genes associated significantly with overall survival.

**Gene**	**HR**	**β**	**95% CI**		***p*-value**
ACADS	0.928366196	−0.00187276	0.865417157	0.995894046	0.038003855
ADRA2A	1.100113247	0.032095812	1.011907879	1.196007246	0.025249061
ALAS1	1.034826363	0.009264945	1.001166195	1.069618218	0.042453742
APOD	1.00111532	0.000186308	1.000420559	1.001810564	0.001649279
ARSF	1.055792449	0.030178285	1.010180463	1.103463922	0.015974629
ESRRB	0.253480421	−0.63080124	0.077106689	0.833291178	0.023801909
FOXO3	1.064472809	0.010691812	1.011461129	1.120262883	0.016520896
HSPH1	1.079350185	0.04288531	1.031190144	1.12975946	0.001042611
KLF15	1.02290405	0.008133916	1.000441435	1.04587101	0.045616945
NR1H4	6.601400413	0.377475499	1.293604889	33.68763353	0.023235624
PCSK1	1.028360599	0.002841669	1.00003015	1.057493639	0.049753259
PIK3R1	1.023174755	0.004369675	1.007451449	1.039143454	0.00373733
RNASEL	1.408182823	0.031593095	1.063799087	1.864053925	0.016748547
RUFY1	1.087608124	0.013758578	1.008466415	1.172960661	0.029355455
SFN	1.123512904	0.060829177	1.046360087	1.206354543	0.001334521
SH3GLB1	1.034549018	0.004449659	1.007253765	1.062583936	0.012782793
SPTSSA	0.97849372	−0.00592499	0.958852265	0.998537518	0.035603438

CI, confidence interval; HR, hazard ratio.

β represented the selected gene coefficient from LASSO analysis.

*P < 0.05 was considered significant statistically*.

### The 17-Gene Risk Signature Shows Strong Prognostic Power

Univariate and multivariate Cox regression analyses were performed to determine prognostic factors for survival in TCGA/CGGA/GSE13041 patients with GBM. As shown in Table 2, the 17-gene risk signature was independently correlated with OS (*p* < 0.05), and the genes signature could also be served as an independent prognostic factor in the CGGA and GSE13041 cohorts (*p* < *0.05*).

**Table 2:** Univariate and multivariate Cox regression analysis of clinical pathologic features for survival of GBM in three datasets (TCGA/CGGA/GEO).

**Variables**	**Univariate cox model**			**Mutivariate cox model**			
	**HR**	**95% CI**	***P-*value**	**Coef**	**HR**	**95% CI**	***P*-value**
***TCGA***							
Age	2.129	1.110–4.082	**2.290E−02**	−1.112E−02	9.889E−01	0.386–2.534	9.815E−01
Gender	0.970	0.669–1.407	8.740E−01				
Subtype	1.231	0.740–2.050	4.510E−01				
IDH1 status	0.253	0.093–0.689	**7.190E−03**	1.422E+01	1.498E+06	0.000–Inf	9.962E−01
MGMT promoter	0.565	0.367–0.872	**9.870E−03**	−3.990E−01	6.710E−01	0.419–1.076	9.741E−02
G-CIMP status	0.168	0.053–0.531	**2.400E−03**	−1.539E+01	2.072E−07	0.000–Inf	9.958E−01
Radiotherapy	0.109	0.026–0.458	**2.520E−03**	−2.324E+00	9.789E−02	0.021–0.441	**2.480E−03**
Chemotherapy	0.453	0.307–0.669	**7.040E−05**	−6.465E−01	5.239E−01	0.314–0.873	**1.305E−02**
Risk Score	2.701	1.843–3.957	**3.430E−07**	7.133E−01	2.041E+00	1.258–3.312	**3.880E−03**
***CGGA***							
Age	1.560	1.113–2.185	**9.780E−03**	0.238	1.269	0.893–1.804	1.841E−01
Gender	0.986	0.722–1.345	9.272E−01				
IDH1 status	0.528	0.339–0.821	**4.610E−03**	−0.733	0.480	0.303–0.760	**1.750E−03**
MGMT promoter	0.899	0.660–1.224	4.990E−01				
1p/19q status	0.729	0.232–2.287	5.880E−01				
Radiotherapy	0.931	0.600–1.445	7.500E−01				
Chemotherapy	0.445	0.309–0.642	**1.460E−05**	−0.962	0.382	0.260–0.561	**9.050E−07**
Risk Score	1.868	1.099–3.175	**2.100E−02**	0.628	1.874	1.058–3.318	**3.125E−02**
***GSE13041***							
Age	1.645	1.152–2.350	**6.200E−03**	0.332	1.394	0.963–2.019	7.862E−02
Gender	0.961	0.708–1.303	7.960E−01				
Subtype	1.850	1.185–2.903	6.323E−02				
Risk Score	1.460	1.078–1.976	**1.440E−02**	0.398	1.488	1.090–2.032	**1.229E−02**

Coef, coefficient; CI, confidence interval; HR, hazard ratio; IDH1, isocitrate dehydrogenase; Inf, Infinite; MGMT, methylguanine methyltransferase.

*P-value (< 0.05) marked in bold was considered significant statistically*.

**Figure 3:**
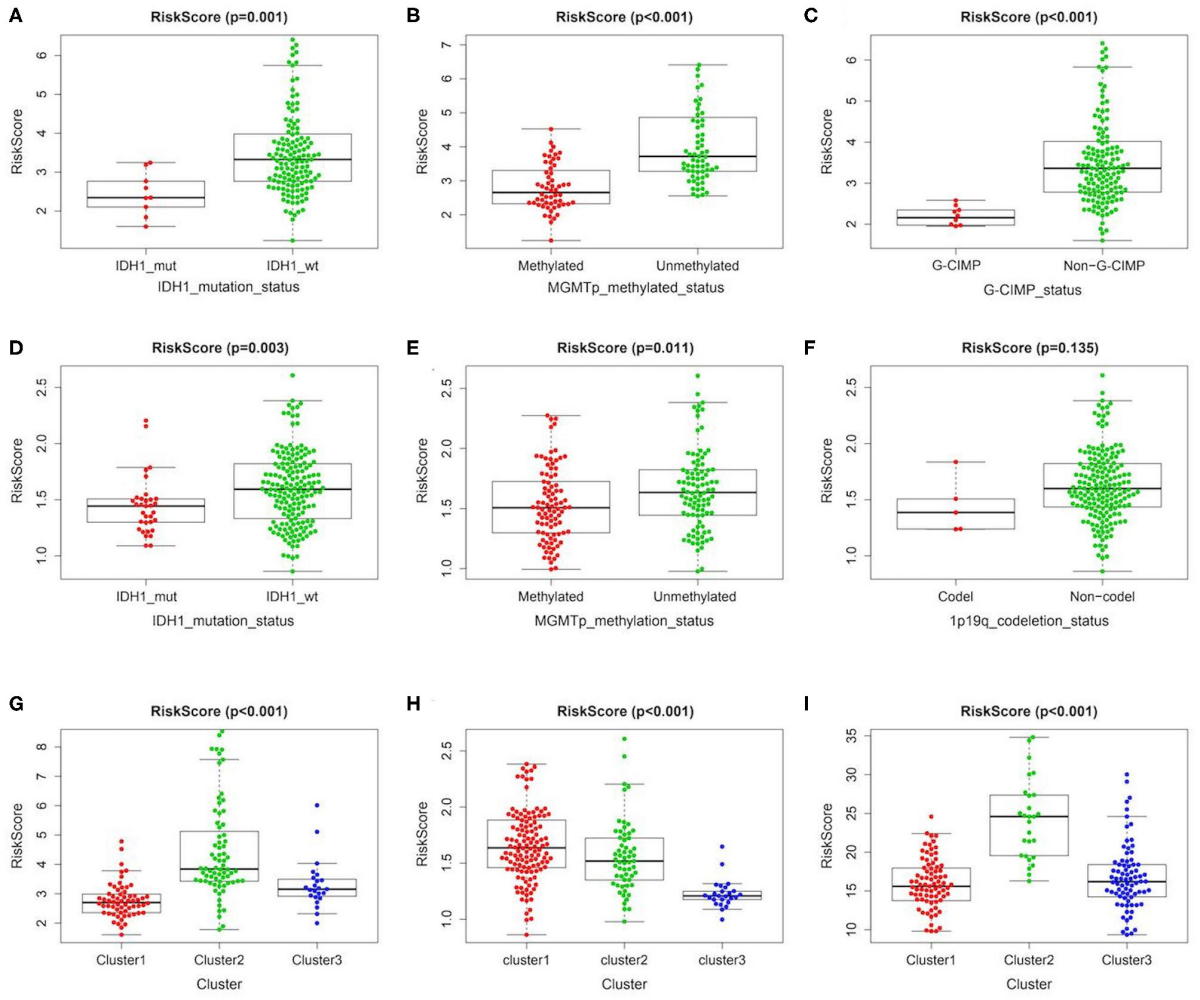
Association between the metabolism-related gene panel and pathologic features. **(A–C)** Distribution of the risk score in stratified patients by IDH1_status, MGMT promoter methylated status and G-CIMP status in TCGA cohort. **(D–F)** Distribution of the risk score in stratified patients by IDH1_status, MGMT promoter methylated status and 1p/19q codeletion status in CGGA cohort. **(G–I)**, Distribution of the risk score among 3 clusters identified of the present study in TCGA, CGGA and GSE13041 cohort, respectively. IDH1, isocitrate dehydrogenase1; MGMT, methylguanine methyltransferase; G-CIMP, glioma cytosine-phosphate-guanine island methylator phenotype.

In order to further clarify the significance of risk signature in GBM, we detected the correlation between risk score and major clinical features. The risk score distribution of patients with different IDH1 status, MGMT promotor methylation status and G-CIMP status was significantly different in TCGA cohort. The risk scores were lower in GBM patients with IDH1-mutant type, MGMT promoter methylated and G-CIMP subgroups than the IDH1-wild type (wt), MGMT promoter unmethylated and non G-CIMP ones of TCGA cohort, respectively (Figures 3A–C). Consistent results could also be observed in the CGGA cohort, the risk scores of IDH1-mutant type and MGMT promoter methylated subgroups were lower than the IDH1-wt and MGMT promoter unmethylated ones, respectively (Figures 3D,E). However, there was no significant difference in risk score between the 1p/19q codel and the non-codel groups, which may be due to the small sample size of the 1p/19q codel group (Figure 3F). In addition, no statistically significant differences were observed between risk scores for different age stratifications and for different molecular subtypes (Supplementary Figure 7). We further compared the differences in risk scores among three different clusters we identified above in TCGA/CGGA/GSE13041. Interestingly, as shown in the Figures 3G–I, the cluster with the worst prognosis had the highest risk score (cluster2 in TCGA and GSE13041, and cluster1 in CGGA), while the cluster with the best prognosis had the lowest risk score (cluster1 in TCGA and GSE13041, cluster3 in CGGA).

**Figure 4:**
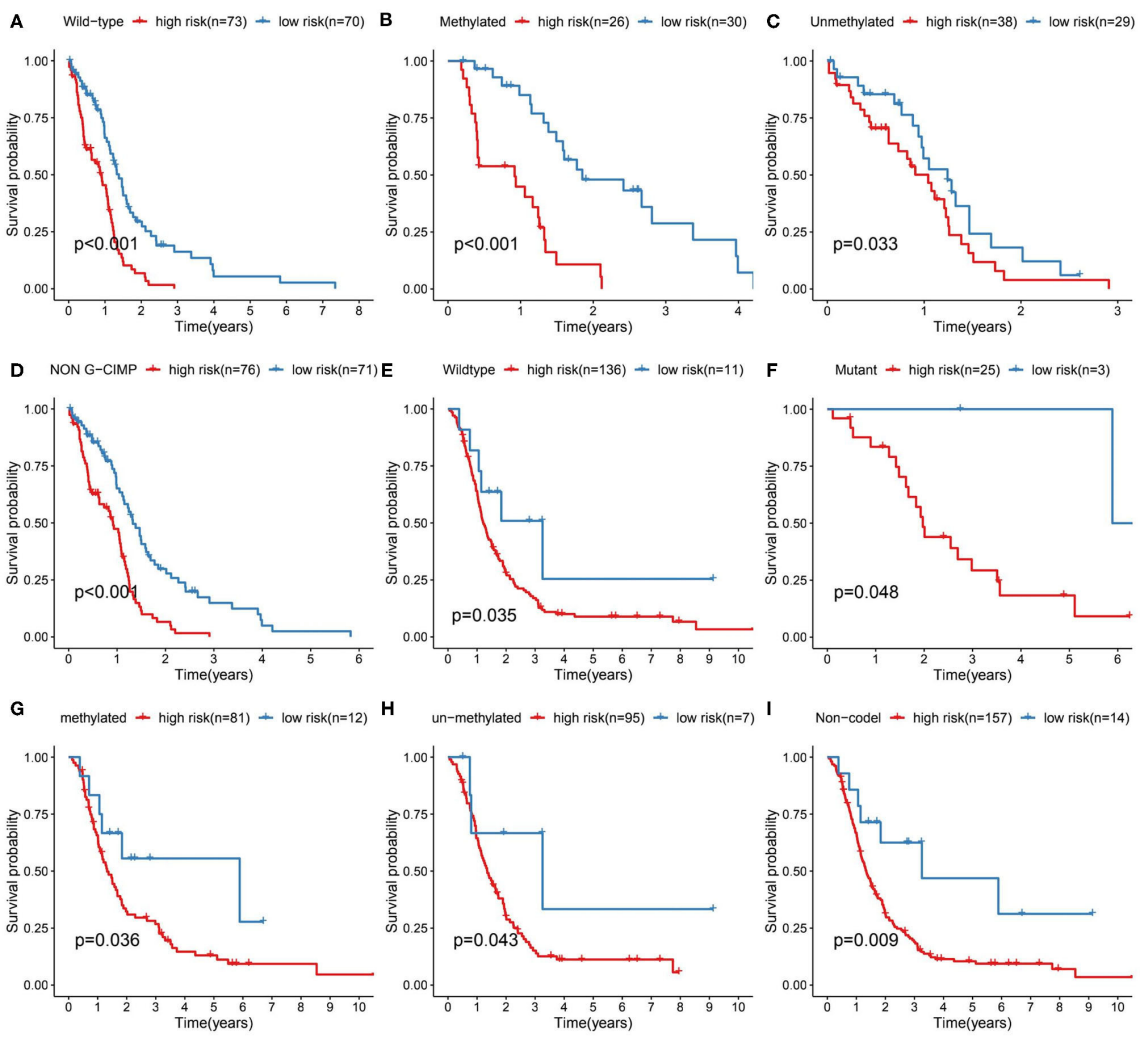
Prediction outcome of the 17-metabolism related gene signature in stratified patients. **(A–D)** Survival analysis of the signature in patients stratified by IDH1wild type, MGMT promoter methylated/unmethylated status and non-G-CIMP status in TCGA cohort. **(E–I)** Survival analysis of the signature in patients stratified by IDH1 wild/mutant status, MGMT promoter methylated/unmethylated status and 1p/19q codeletion status in CGGA cohort. IDH1, isocitrate dehydrogenase1; MGMT, methylguanine methyltransferase; G-CIMP, glioma cytosine-phosphate-guanine island methylator phenotype.

We further validated the prognostic predictive power of the risk signature we identified in different clinical feature stratified groups, such as IDH1-wt/-mutant, MGMT promoter methylated/unmethylated, G-CIMP/non G-CIMP, and 1p/19q codel/non-codel cohorts. In the TCGA and CGGA cohorts, KM analysis showed that cases with high-risk score had shorter OS than the low-risk ones in most stratified patients (Figure 4). However, this result was not observed in the TCGA_IDH1-mutant group, TCGA_G-CIMP group and CGGA_ 1p/19q-codel group due to the small sample size of cases.

**Figure 5:**
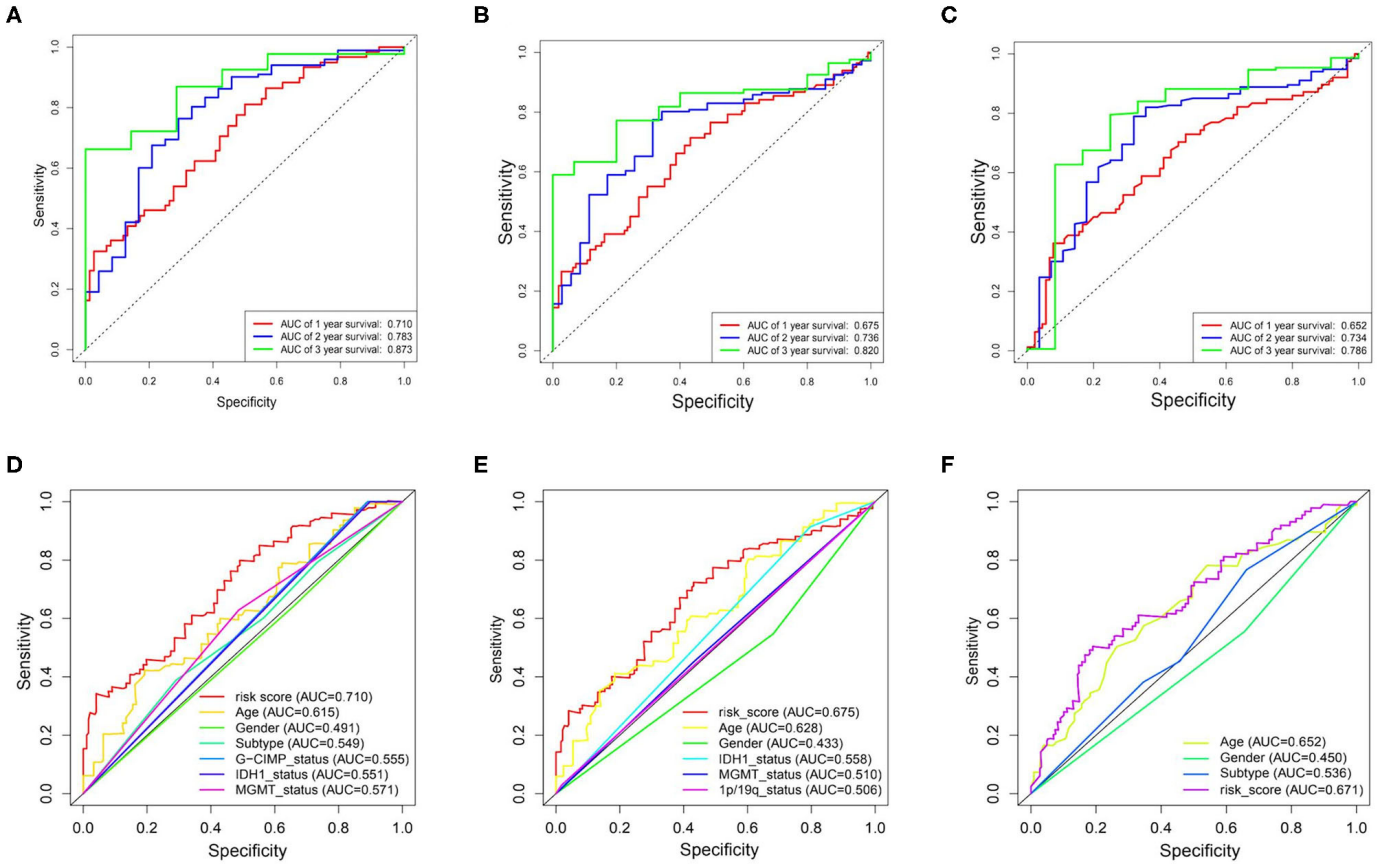
Prognostic power of the identified 17-gene signature in TCGA/CGGA/GSE13041 cohorts. **(A–C)**, ROC analyses in TCGA, CGGA, and GSE13041 cohorts for 1-, 2-, and 3-year. **(D)** ROC curve analysis of age, gender, subtype, IDH1_status, MGMT_status, G-CIMP_status, and risk score in TCGA cohort. **(E)** ROC curve analysis of age, gender, IDH1_status, MGMT_status, 1p/19q_status, and risk score in CGGA cohort. **(F)** ROC curve analysis of age, gender and risk score in GSE13041 cohort. AUC, area under the curve; ROC, receiver operating characteristic; IDH1, isocitrate dehydrogenase1; MGMT, methylguanine methyltransferase; G-CIMP, glioma cytosine-phosphate-guanine island methylator phenotype.

By performing the ROC analysis in the training datasets and validation datasets, we next evaluate the accuracy of the risk signature. The result showed that the AUC value of 1-, 2-, and 3-year for the TCGA datasets were 0.710, 0.783, and 0.873, respectively (Figure 5A). And in the CGGA/GSE validation sets, a similar strong prognostic power was obtained (Figures 5B,C). In addition, we also compared the accuracy of the clinical features and risk score for the survival prediction of GBM and discovered that our risk signature is the optimal (Figures 5D–F). These results all confirmed that the metabolism risk signature we constructed had a strong prognostic prediction power.

### The High- and Low-Risk Groups Present Different Biologic Processes

**Figure 6:**
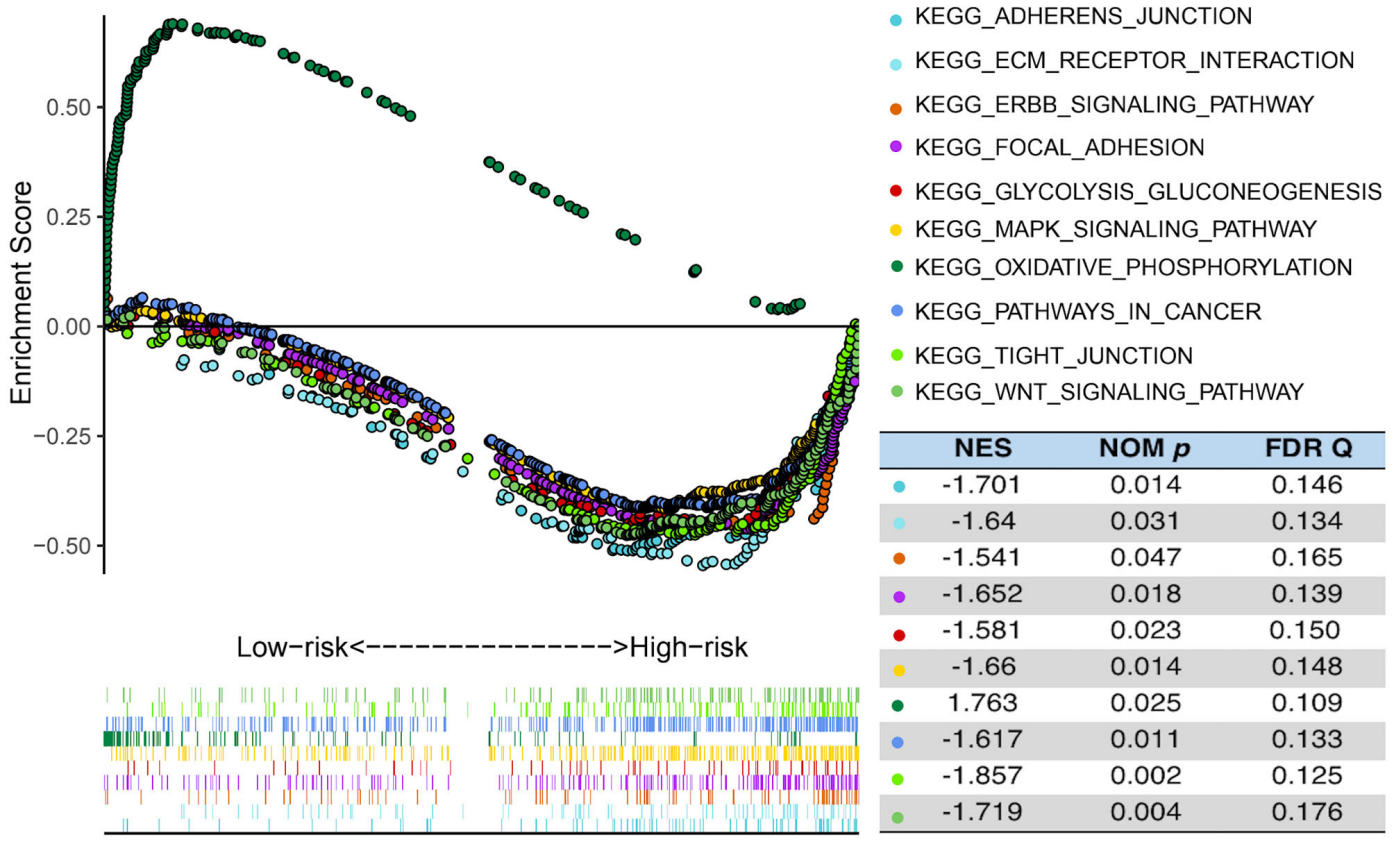
Functional enrichments between high- and low-risk cases of TCGA. GSEA analysis based on the median value of the risk score in TCGA. FDR, false discovery rate; NES, normalized enrichment score; Nom, nominal.

In order to explore the difference in biological process between the high- and the low-risk group in the TCGA cohort, GSEA was performed to compare the gene expression of patients in the two groups. PCA showed that in the three public cohorts of TCGA/CGGA/GSE13041 cases in the high-risk and low-risk groups were significantly distributed in two regions (Supplementary Figure 8). As shown in the Figure 6, GSEA indicated that glycolysis gluconeogenesis, WNT signaling pathway, pathways in cancer, MAPK signaling pathway, ERBB signaling pathway, adherens junction, focal adhesion, ECM receptor interaction and tight junction were significantly enriched in high-risk patients, while low-risk cases showed enrichment of OxPhos. The significantly pathways were selected based on the screening criteria of nominal *p* < 0.05 and false discovery rate (FDR) <0.25. The results indicated that there were significant differences in biological processes between the two groups of GBM with different metabolic risk levels.

### CMap Analysis Identifies Novel Candidate Compounds Targeting the GBM Clusters

**Figure 7:**
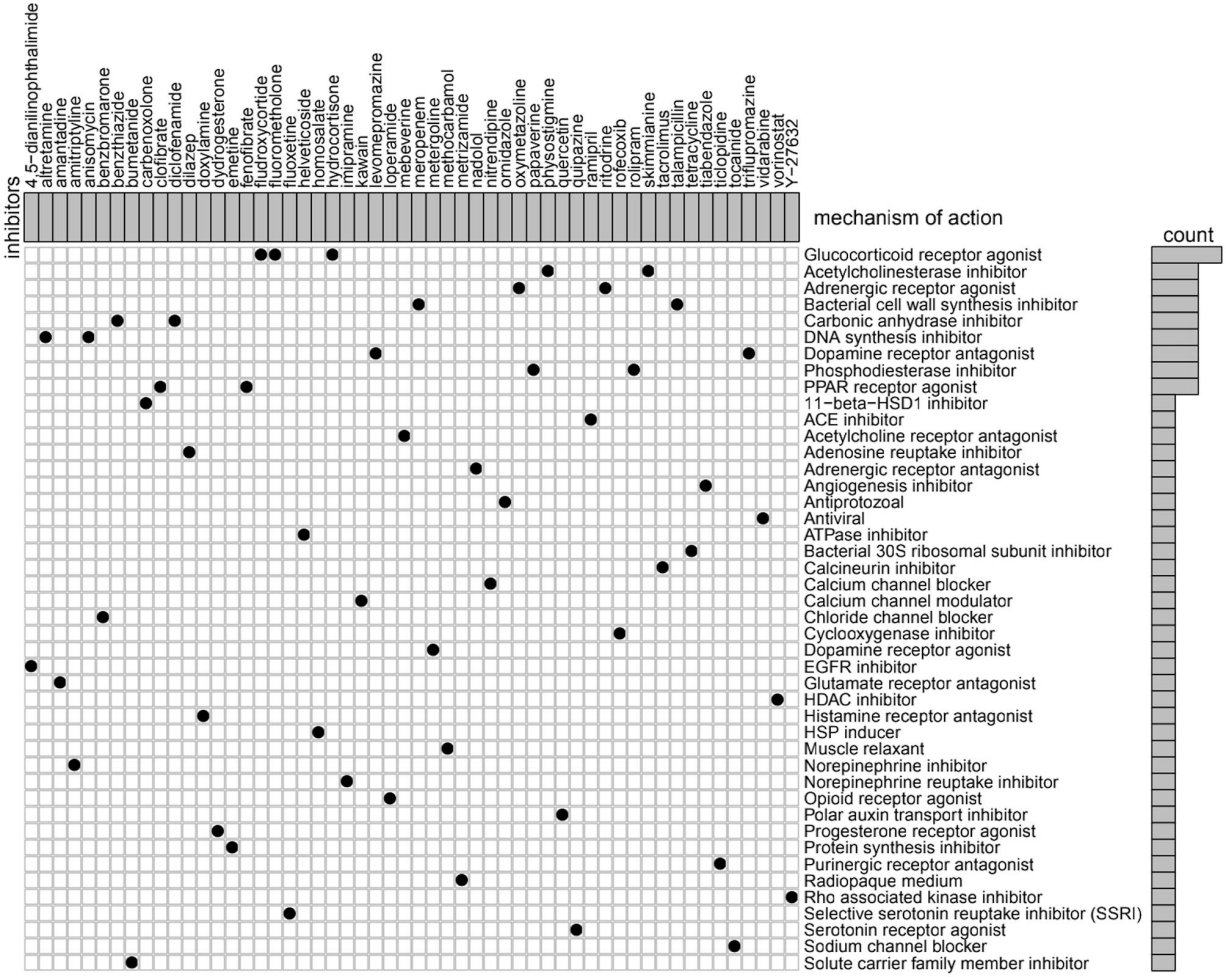
Heatmap showing each compound (perturbagen) from the CMap that shares a MoA (rows), sorted by descending number of compounds with a shared MoA. The above compounds have an absolute value of enrichment score ≥ 0.5 and might be capable of targeting the metabolism-related signature. CMap, Connectivity Map; MoA, mode of action.

To identify potential compounds capable of targeting the pathways associated with metabolism-related genes, we queried the CMap database using the mRNA expression signatures by applying differential expression analysis to GBM subgroups samples. The 54 compounds that were able to repress the above gene expression profile of GBM are shown in Figure 7. CMap mode of action (MoA) analysis of the 54 compounds revealed 44 mechanisms of action shared by the above compounds. Three compounds (fludroxycortide, fluorometholone, and hydrocortisone) shared the MoA of glucocorticoid receptor agonist, and two compounds (physostigmine and skimmianine) shared the MoA of acetylcholinesterase inhibitor. Moreover, a total of 14 compounds shared the following 7 mechanisms: adrenergic receptor agonists, inhibitors of bacterial cell wall synthesis, carbonic anhydrase inhibitors, DNA synthesis inhibitor, dopamine receptor antagonist, and phosphodiesterase inhibitors.

## Discussion

In the present study, through the comprehensive analysis of genes involved in glucose, lipid, and glutamine metabolism of GBM patients in three datasets, the risk signature closely related to the prognosis of GBM was constructed, and the heterogeneity of GBM metabolism was further revealed. Firstly, leveraging a large cohort of GBM profile, 3 clusters of GBM with different clinical features were successfully obtained through the cluster analysis. Subsequent analysis revealed that the 3 clusters had significantly different risk scores. In addition, we identified a 17 metabolism-related gene risk signature as a significant independent predictor for the prognosis of GBM by univariate cox regression analysis and LASSO analysis. GSEA suggested glycolysis gluconeogenesis and OxPhos were significantly enriched in high- and low-risk GBM. Moreover, we further demonstrated the robustness of molecular subtype and the predictive power of 17 metabolism-related gene risk signature in two validation datasets, CGGA and GSE13041, and achieved consistent results. Finally, we used CMap database to screen compounds that may target metabolism-related genes, and it is hoped that targeted therapy can be performed on GBM clusters with different metabolic status.

Considering that univariate Cox model has insufficient dimensional data on variable selection, we first performed univariate Cox model to obtain the genes related to overall survival and applied Cox LASSO regression to improve the performance index for predicting prognosis (26). None of the 17 genes showed high coefficients in the Cox model, but the cumulative effect of the 17 genes signature on OS obtained the optimal survival prediction. Interestingly, in the 17-metabolism gene risk signature we constructed, it has been reported that *FOXO3* acetylation plays a central role in the regulation of glycolytic metabolism in glioblastoma, and the survival of GBM patients with *FOXO3* acetylation is shorter (27). In addition, *FOXO3* is related to GBM temozolomide (TMZ) resistance, and the phosphorylated AKT/FOXO3 axis regulates the expression of long non-coding RNA related to TMZ resistance GBM cells (28). Another gene, *PIK3R1*, is part of the RTK/ RAS/(3)K signaling pathway, which is mutated in many cancers and plays a key role in the proliferation, differentiation, and survival of cancer cells. Nearly 90% of normal GBMs showed different degrees of *PIK3R1* changes, leading to abnormal activation of RTK/RAS/PI(3)K signal cascade (29). *PIK3R1* has been found to promote the transformation of malignant astrocytes into glioma-like state (30). Stratifin (*SFN*, 14-3-3 sigma), as an oncogene related to cell proliferation, facilitates the development and progression of a variety of cancers (31, 32) including gliomas (33) and has the potential to be a new therapeutic target. In addition, *APOD* has been identified to be associated with astrocytoma progression (34). And *SH3GLB1*, as the autophagy-related gene, is associated with glioma prognosis. Knockdown of *SH3GLB1* inhibits glioma cell proliferation, migration and invasion, and improves sensitivity to temozolomide (35). In addition, some of the genes in the 17-metabolism risk signature are involved in the development of a variety of cancers. *NR1H4*, also known as farnesoid X receptor (*Fxr*), is the gene with the highest positive coefficient and hazard ratio in the metabolism-related risk signature. Previous studies have shown that NR1H4 plays an important role in the development of colon cancer by regulating the stability of *c-Myc* (36). *KLF15* has been reported to inhibit cell growth in lung adenocarcinoma (37), gastric cancer (38), and colorectal cancer (39), and can be used to predict prognosis. High *ADRA2A* expression was associated with poor overall survival for breast (40) and bladder cancer (41). As a genetic marker, *RNASEL* has been linked to lethal prostate cancer (42). And *ESRRB* (or *ERR*β) is a negative regulator of cell cycle and may be a therapeutic target for breast cancer (43). Inhibition of *HSPH1* downregulates the expression of Bcl-6 and *c-Myc* and hampers the growth of human aggressive B cell non-Hodgkin lymphoma (44). *ACADS*, as one of the key metabolic genes related to the metabolic response involved in carcinogenesis, is regulated by DNA methylation and can be used as a potential methylation biomarker related to the proliferation and metastasis of hepatocellular carcinoma (45). *RUFY1*, named RUN, and FYVE domain containing 1, is a member of *RUFY* family (46). *RUFY1* regulates the transport of integrins and participates in the migration of NIH-3T3 fibroblasts (47). Evidence shows that *RUFY1*, as a tumor promoter gene, plays an important role in the development of gastric cancer. Targeting *PODXL/RUFY1* complex may improve the prognosis of gastric cancer (GC) and provide new treatment opportunities for GC patients (48). Studies have shown that *PCSK1* is the gene most significantly associated with poor response to concurrent chemoradiotherapy (CCRT) in rectal cancer. Therefore, overexpression of *PCSK1* is one of the risks of poor CCRT response and prognosis in rectal cancer patients (49). And *PCSK1* is also over expressed in pure fibrolamellar hepatocellular carcinoma as one of neuroendocrine genes (50). As for *ALAS* gene, in non-small-cell lung cancer, non-erythrocyte *ALAS1* gene transcription levels and *ALAS1* protein levels were significantly elevated in cancer cells, while *ALAS2* transcription levels were increased nearly 5-fold in HCC4017 cells (51). However, 2 of the 17-metabolism risk genes, including *SPTSSA and ARSF* have not been studied in cancers. *SPTSSA*, also known as serine palmityl transferase small subunit A (*SPTSSA*), encodes A subunit of SPT that is a rate-limiting enzyme in the sphingolipid biosynthesis pathway (52). As a component of eukaryotic membranes, SLs has a variety of critical functions in the growth and development of embryos and the maintenance of normal physiology (53). In a clear cell renal cell carcinoma (ccRCC) study, bioinformatics analysis revealed that 10 genes, including *SPTSSA*, significantly coregulated ccRCC with *SPTLC1*, and the low expression of *SPTLC1* was significantly correlated with the disease progression and poor prognosis of ccRCC (54). In addition, another gene *ARSF*, which is located in Xp22.3 with *ARSD* and *ARSE*, has significant homology and highly similar intron/exon structure. Meanwhile, the splicing sites of *ARSF, ARSD, ARSE*, and *ARSC* are all at the same position. The biological role of *ARSF* may therefore be masked by the other three genes (55). The biological role of 17 metabolism-related genes in glioblastoma remains to be further explored.

The Warburg effect has been widely described in GBM and other tumor types. According to the Warburg hypothesis (14), cancers are partly caused by impaired mitochondrial function and OxPhos, which is characterized by cancer cells producing most of their energy through glucose fermentation (such as aerobic glycolysis), with limited OxPhos capacity (56). This metabolic reprogramming is thought to be an adaptive mechanism for the rapid growth of tumor cells to meet their increasing energy needs. However, the intrinsic cellular heterogeneity of GBM raises the question of whether the survival and proliferation of different cell subsets is limited to glucose fermentation or other metabolic pathways. Recent studies suggest that the residual activity of mitochondrial function in GBM cells can still provide OxPhos for cancer cells (57–59). According to Deleyrolle LP's study (6), subgroups of cells with different metabolic requirements exist in GBM, in which fast-cycling cells utilize aerobic glycolysis, while slow-cycling cells preferentially utilize mitochondrial OxPhos to obtain metabolism energy. But some studies hold the opposite view that GBM tissue are unable to obtain significant energy from OxPhos. Because ultrastructural and biochemical evidence suggests that GBM cells exhibit defects in the number, structure and function of mitochondria, thus incompatible with OxPhos energy production (60–63). In addition, large numbers of mitochondrial DNA (mtDNA) mutations have been found in 13 cancers including GBM that compromise OxPhos function (64). Therefore, emerging evidence indicates that cancer cells including GBM obtain energy through the glutaminolysis pathway using mitochondrial substrate-level phosphorylation (mSLP) as an alternative to OxPhos (61). Moreover, some studies based on the above theory, ketogenic metabolic therapy, as an alternative standard of care, has the potential to improve outcomes for GBM patients and other malignant brain cancers, and has yielded impressive results in clinical practice for GBM treatment (65–69). Here, functional analysis in our study indicated that GBMs are metabolic heterogeneity and the biological process differences between the high- and the low-risk group mainly focused on the metabolic patterns and signaling pathways. Glycolysis gluconeogenesis and OxPhos were significantly enriched in high- and low-risk GBMs. Whether OxPhos is caused by mSLP remains to be confirmed by future studies. In addition, our functional enrichment analysis identified metabolic signaling pathways associated with high-risk GBM, including WNT signaling pathway, MAPK signaling pathway, ERBB signaling pathway and pathways in cancer. Of which, abnormal Wnt signaling is recognized to drive metabolic alterations such as glycolysis, lipogenesis and glutaminolysis, which are critical to the survival of cancer stem cells (70). The pathogenesis of GBM involves multiple levels of WNT signal pathway, including tumorigenesis, stem cell maintenance, invasion, and drug resistance. Inhibition of WNT signal transduction is expected to be a new direction of GBM therapy (70, 71).

CMap is a systematic tool that uses gene-expression signatures to probe the relationship between small molecules, genes and disease (72). It screens new treatments for various diseases by comparing changes in gene expression or signature caused by disease, genetic perturbation (knockdown or overexpression of genes) or small molecule therapy with the similarity of all perturbation signatures in the database. In this study, we used CMap analysis to accurately identify compounds that have been shown to have specific effects on GBM or other tumor types by comparing the different expression genes of GMB samples from 3 clusters. These compounds include the DNA synthesis inhibitor anisomycin (73), glutamate receptor antagonist amantadine (74), norepinephrine inhibitor amitriptyline (75, 76), solute carrier family member inhibitor bumetanide (77), PPAR receptor agonist clofibrate (78), and fenofibrate (79), selective serotonin reuptake inhibitor (SSRI) fluoxetine (75, 80), ATPase inhibitor helveticoside (81, 82), norepinephrine reuptake inhibitor imipramine (75, 76, 83, 84), opioid receptor agonist loperamide (85), phosphodiesterase inhibitor papaverine (86, 87) and rolipram (88–92), polar auxin transport inhibitor quercetin (93, 94), cyclooxygenase inhibitor rofecoxib (95, 96), calcineurin inhibitor tacrolimus (97), purinergic receptor antagonist ticlopidine (98, 99), HDAC inhibitor vorinostat (100), Rho associated kinase inhibitor Y-27632 (101–103).

Interestingly, our results suggest that some conventional antipsychotics among drugs mentioned above, including amitriptyline, fluoxetine, and imipramine, may also play a surprising role in the treatment of GBM, which is consistent with previous studies and offers new hope for patients with GBM (12, 75, 76, 80, 83, 84, 104). In addition, Leite et al. (105) demonstrated that clomipramine (tricyclic antidepressant drugs such as imipramine) has an impact on GBM growth and has no toxicity in normal cells (astrocytes and microglia). Reaserches have further elucidated the potential mechanism of conventional antidepressants therapy for GBM. By targeting the respiratory chain complex III and changing membrane potential, the tricyclics antidepressants mentioned above induce mitochondria-mediated apoptosis of malignant glioma cells, activate the intrinsic pathway of cytochrome-C release and caspase-3 dependent apoptosis process, and finally results in glioma cell death (106–112). It is worth mentioning that previous studies have also indicated that the anticoagulant ticlopidine synergized with imipramine to induce the death of human glioma cell lines and primary mouse glioma cells (98, 99). In addition, some of the other drugs in our findings mentioned above have been reported as a combination of glioblastoma chemotherapy, such as rollipram, a promising immunotherapeutic adjuvant that can potentiate the effect of bevacizumab on GBM (89, 90). The antiangiogenic effect of rofecoxib makes GBM chemotherapy more effective (96). Tacrolimus endows the GBM stem-like cells chemosensitive to the MRP1 drug substrate (97). Of course, it is still controversial whether some of the drugs identified by our study are effective against GBM. Such as vorinostat combined with bevacizumab significantly inhibited angiogenesis of GBM stem cells *in vitro* (100), but no significant benefit was observed in patients with GBM in clinical trials (113). However, our study provides evidence to explore the underlying mechanism of these drugs in the treatment of GBM, and it may be a new direction to investigate whether these drugs have the potential to treat GBM from a metabolic perspective.

However, some limitations of our present study should be acknowledged. First, GSE13041, one of the external validation datasets which was selected in this study lacked some clinical information, so the prognostic value of signatures needed to be validated by more external datasets, and multicenter prospective studies are needed to assess the feasibility of risk gene signatures in the future. Second, the deep molecular mechanisms behind our findings need to be further elucidated in experimental studies to facilitate our understanding of the functional roles and clinical applications of metabolism-related genes and potential molecular compounds. In particular, the issue of whether GBM tumor cells can utilize Oxphos (or mSLP) to generate the required energy remains to be further explored due to the lack of support from relevant datasets.

## Conclusion

Our study identified a novel metabolism-related gene signature, in addition the existence of three different metabolic status and two opposite biological processes in GBM were recognized, which revealed the metabolic heterogeneity of GBM. Robust metabolic subtypes and powerful risk prognostic models contributed a new perspective to the metabolic exploration of GBM.

## Data Availability Statement

The datasets analyzed for this study are available from the Cancer Genome Atlas (TCGA) (http://cancergenome.nih.gov/), the Chinese Glioma Genome Atlas (CGGA) (http://www.cgga.org.cn/index.jsp), and Gene Expression Omnibus (GEO) (https://www.ncbi.nlm.nih.gov/geo/query/acc.cgi?acc=GSE13041).

## Author Contributions

GL and HX conceived and designed the study. RZ put forward constructive suggestions to the study. ZH and CW analyzed the results, completed the visualization of the image, and contributed equally to this work. ZH completed the manuscript. All authors participated in the preparation approved the final manuscript.

## Conflict of Interest

The authors declare that the research was conducted in the absence of any commercial or financial relationships that could be construed as a potential conflict of interest.
