# Supplementary material for: Identification of a Metabolism-Related Risk Signature Associated With Clinical Prognosis in Glioblastoma Using Integrated Bioinformatic Analysis
Source: Front Oncol. 2020 Sep 3;10:1631. doi: 10.3389/fonc.2020.01631 (PMC7523182; doi:10.3389/fonc.2020.01631)
Supplement: Supplementary file 1 [file Data_Sheet_1.docx]

Supplementary Material

# Supplementary Tables

**Supplementary Table S1. Basic clinical and molecular characteristics of the three public datasets.**

| **Variables** | | **TCGA** | **CGGA** | **GSE13041** | |
| --- | --- | --- | --- | --- | --- |
|  |  | **(n=165)** | **(n=216)** | **(n=191)** | |
| **Age** | ＜45 yrs | 21 | 69 | 53 | |
|  | ≥45 yrs | 144 | 147 | 138 | |
| **Gender** | Male | 107 | 181 | 117 | |
|  | Female | 58 | 124 | 74 | |
| **IDH1_status** | Mut | 9 | 29 | 0 | |
|  | WT | 150 | 183 | 0 | |
|  | NA | 6 | 4 | 191 | |
| **1p/19q_codel status** | Codel | 0 | 5 | 0 | |
|  | Non codel | 165 | 171 | 0 | |
|  | NA | 0 | 40 | 191 | |
| **G-CIMP_status** | G-CIMP | 10 | 0 | 0 | |
|  | Non G-CIMP | 146 | 0 | 0 | |
|  | NA | 10 | 216 | 191 | |
| **MGMT promoter_status** | Methylated | 58 | 93 | 0 | |
|  | Unmethylated | 67 | 102 | 0 | |
|  | NA | 40 | 21 | 191 | |
| **Subtype** | Classical | 39 | 0 | Pro | 48 |
|  | Mensenchymal | 52 | 0 | Mes | 69 |
|  | Proneural | 46 | 0 | PN | 56 |
|  | Neural | 28 | 0 | ProMes | 18 |
|  | NA | 0 | 215 | 0 | |
| **Radiotherapy** | Yes | 67 | 145 | 23 | |
|  | No | 93 | 51 | 0 | |
|  | NA | 5 | 20 | 168 | |
| **Chemotherapy** | Yes | 97 | 158 | 20 | |
|  | No | 63 | 43 | 0 | |
|  | NA | 5 | 15 | 171 | |

Abbreviations: IDH1,isocitrate dehydrogenase1; G-CIMP, glioma cytosine-phosphate-guanine island methylator phenotype; MGMT,methylguanine methyltransferase; Mut, mutant; WT, wild type；codel, codeletion; PN, ProNeural, Pro, Proliferative; Mes,Mesenchymal；ProMes, Pro and Mes.

# Supplementary Figures

**
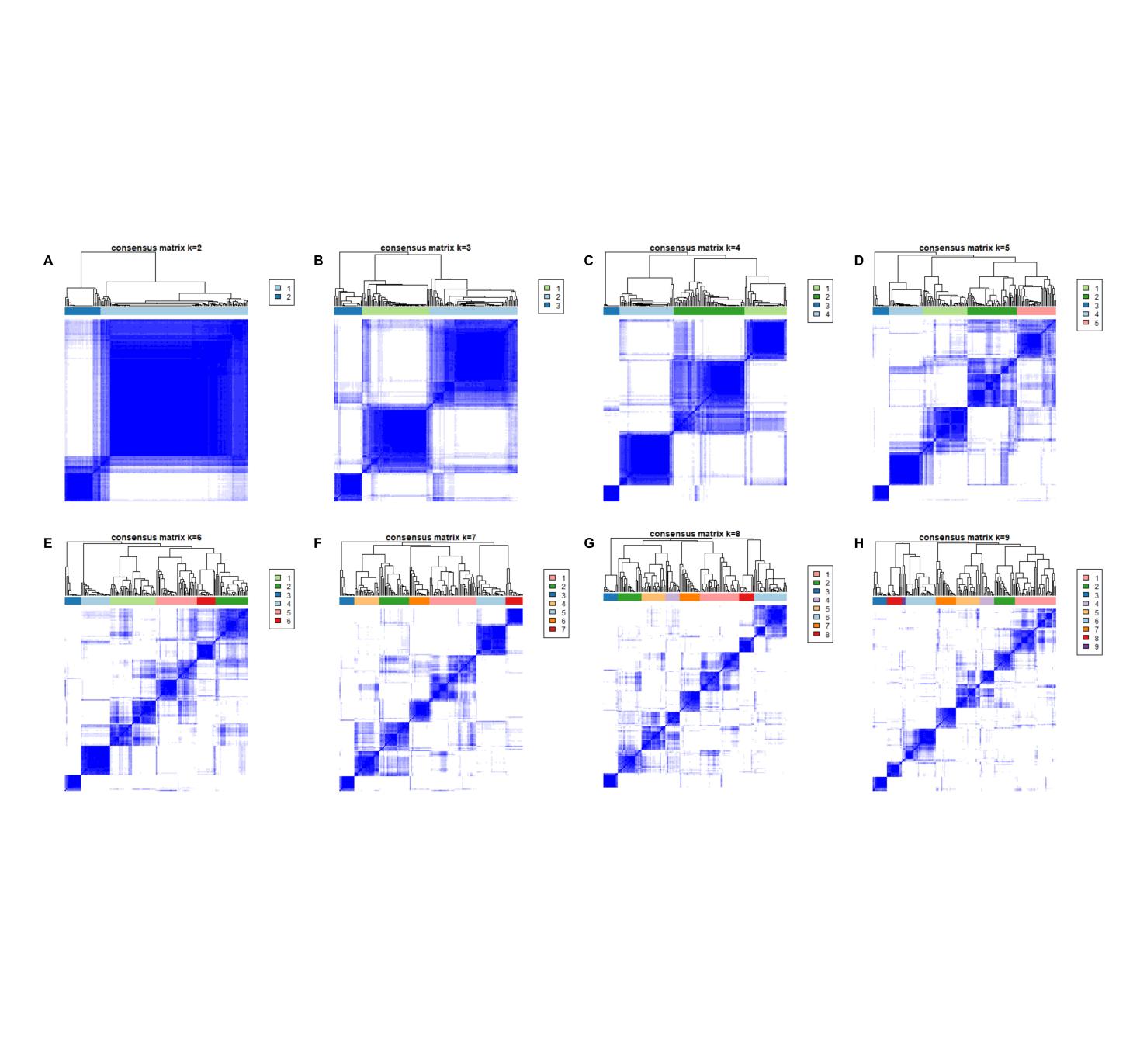
**

**Supplementary Figure S1.** **(A)-(H)** Consensus matrixes for k=2 to k=9 of the 165 patients in the TCGA datasets by clustering the gene expression profile of the 1395 metabolism-related genes.


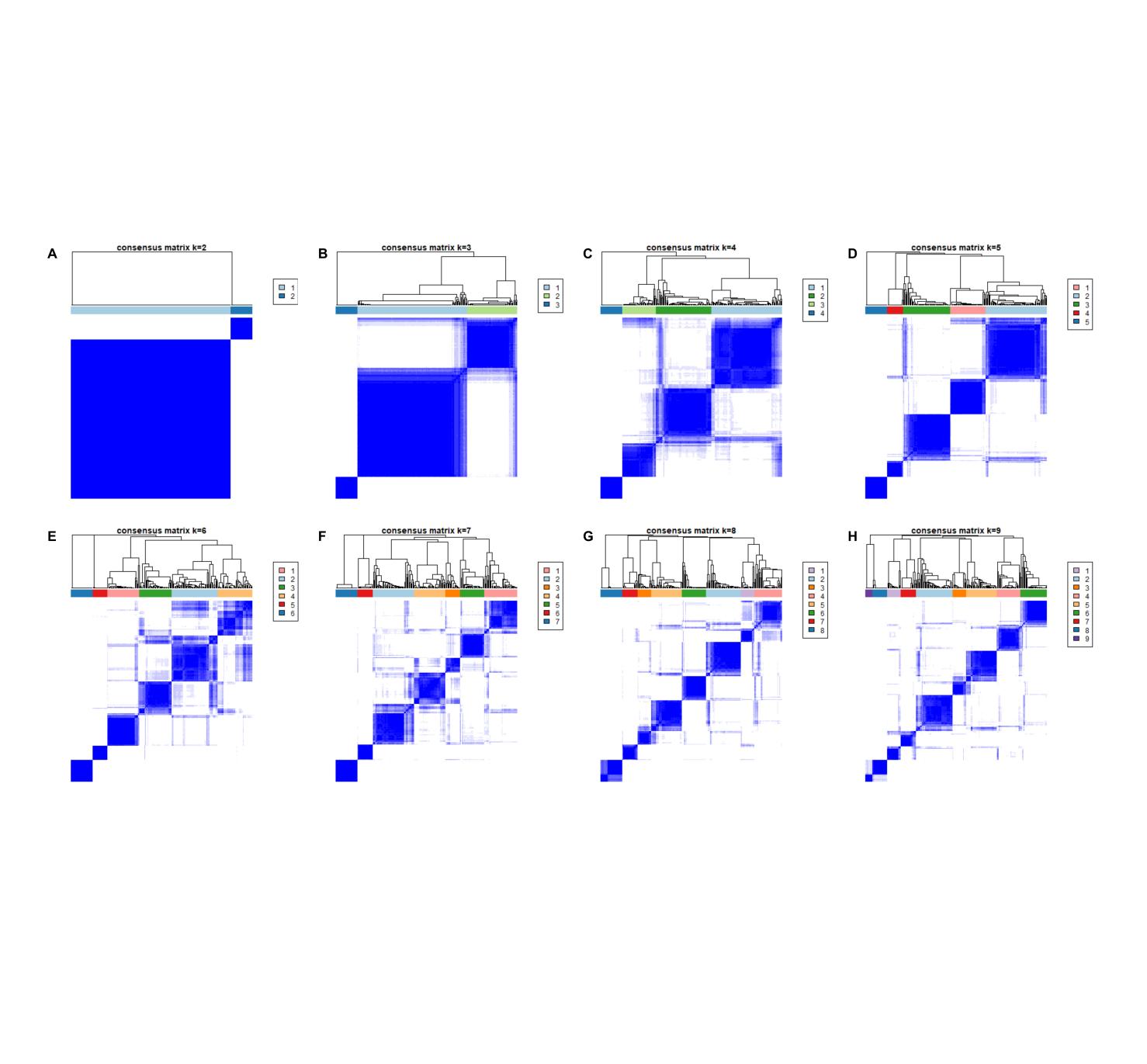


**Supplementary Figure S2.** **(A)-(H)** Consensus matrixes for k=2 to k=9 of the 216 patients in the CGGA datasets by clustering the gene expression profile of the 1395 metabolism-related genes.


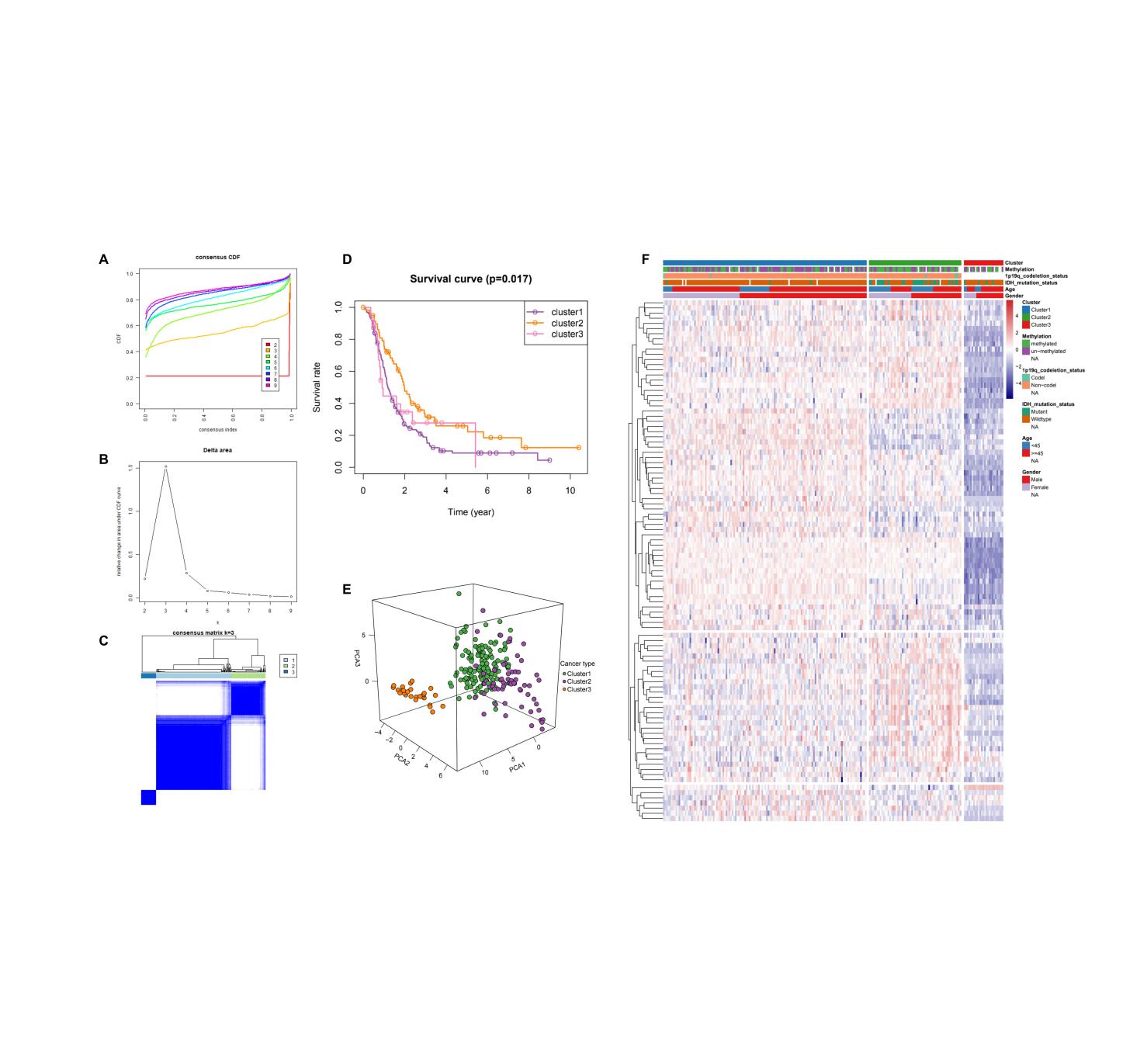


**Supplementary Figure S3.** Metabolism-related genes could distinguish GBM patients in CGGA with different clinical and molecular features. **(A)** Consensus clustering CDF for k = 2 to k = 9. **(B)** Relative change in area under CDF curve for k = 2 to k = 9. **(C)** Consensus clustering matrix heatmap plots of 216 samples from CGGA datasets for k = 3. **(D)** Kaplan-Meier analysis of patients among 3 clusters. **(E)** PCA analysis of the metabolism-related genes expression when k =3. **(F)** Heatmap of three clusters defined by the top 100 variable expression genes. CDF, cumulative distribution function; PCA, principal components analysis.


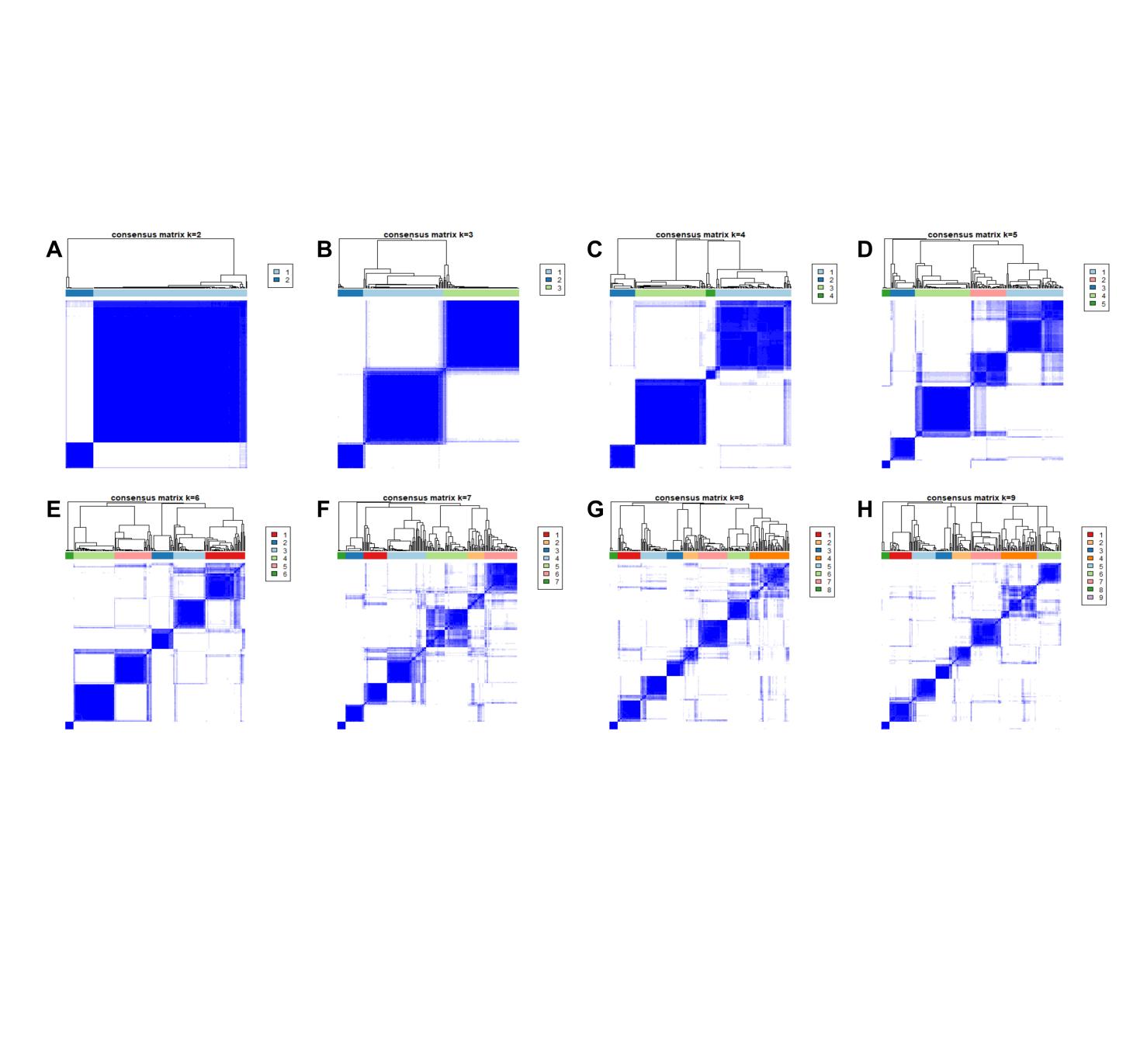


**Supplementary Figure S4.** Consensus matrixes for k=2 to k=9 of the 191 patients in the GSE13041 datasets by clustering the gene expression profile of the 1395 metabolism-related genes.


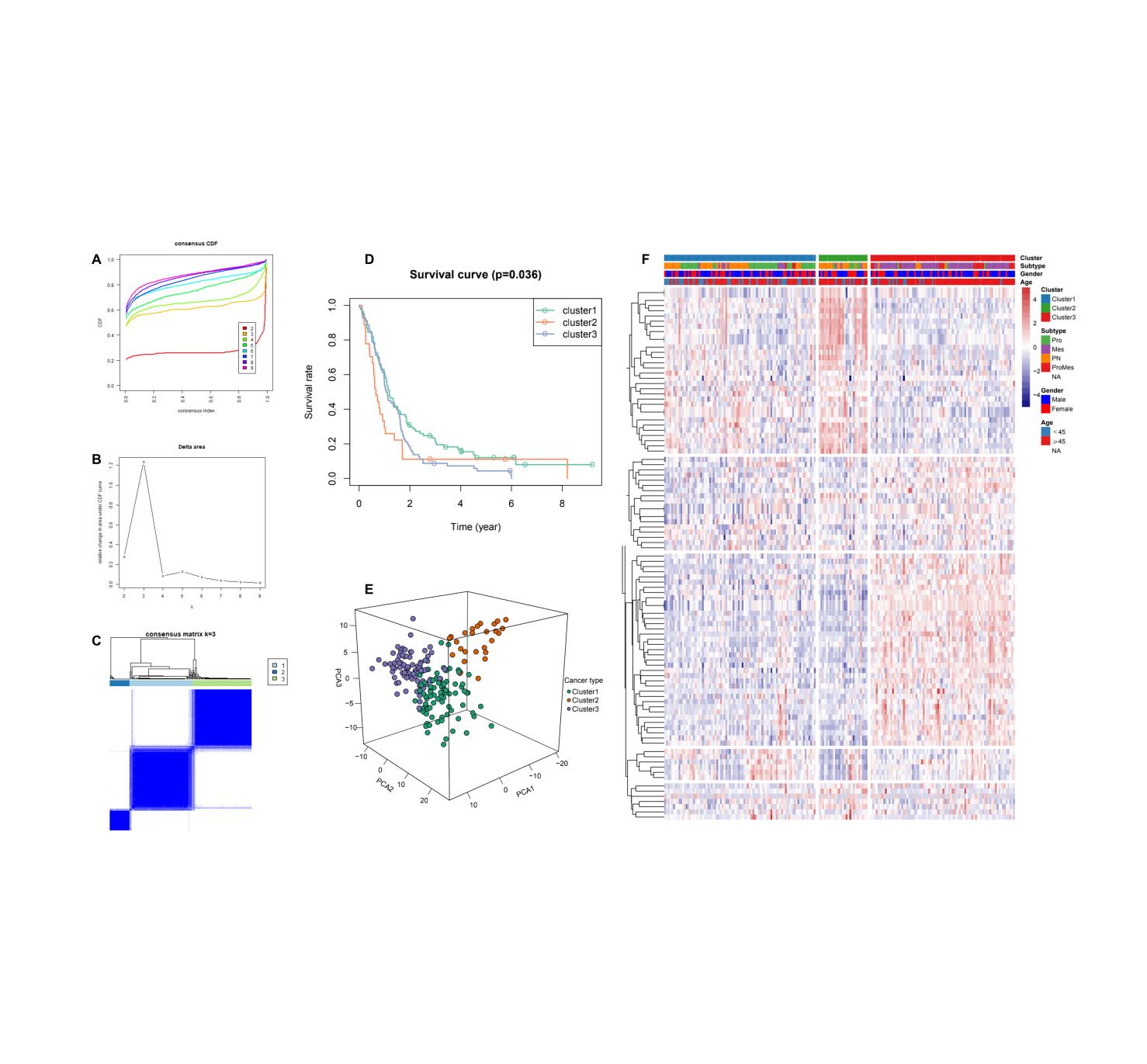


**Supplementary Figure S5.** Metabolism-related genes could distinguish GBM patients in GSE13041 with different clinical and molecular features. (A) Consensus clustering CDF for k = 2 to k = 9. (B) Relative change in area under CDF curve for k = 2 to k = 9. (C) Consensus clustering matrix heatmap plots of 191 samples from GSE13041 datasets for k = 3. (D) Kaplan-Meier analysis of patients among 3 clusters. (E) PCA analysis of the metabolism-related genes expression when k =3. (F) Heatmap of three clusters defined by the top 100 variable expression genes. CDF, cumulative distribution function; PCA, principal components analysis.


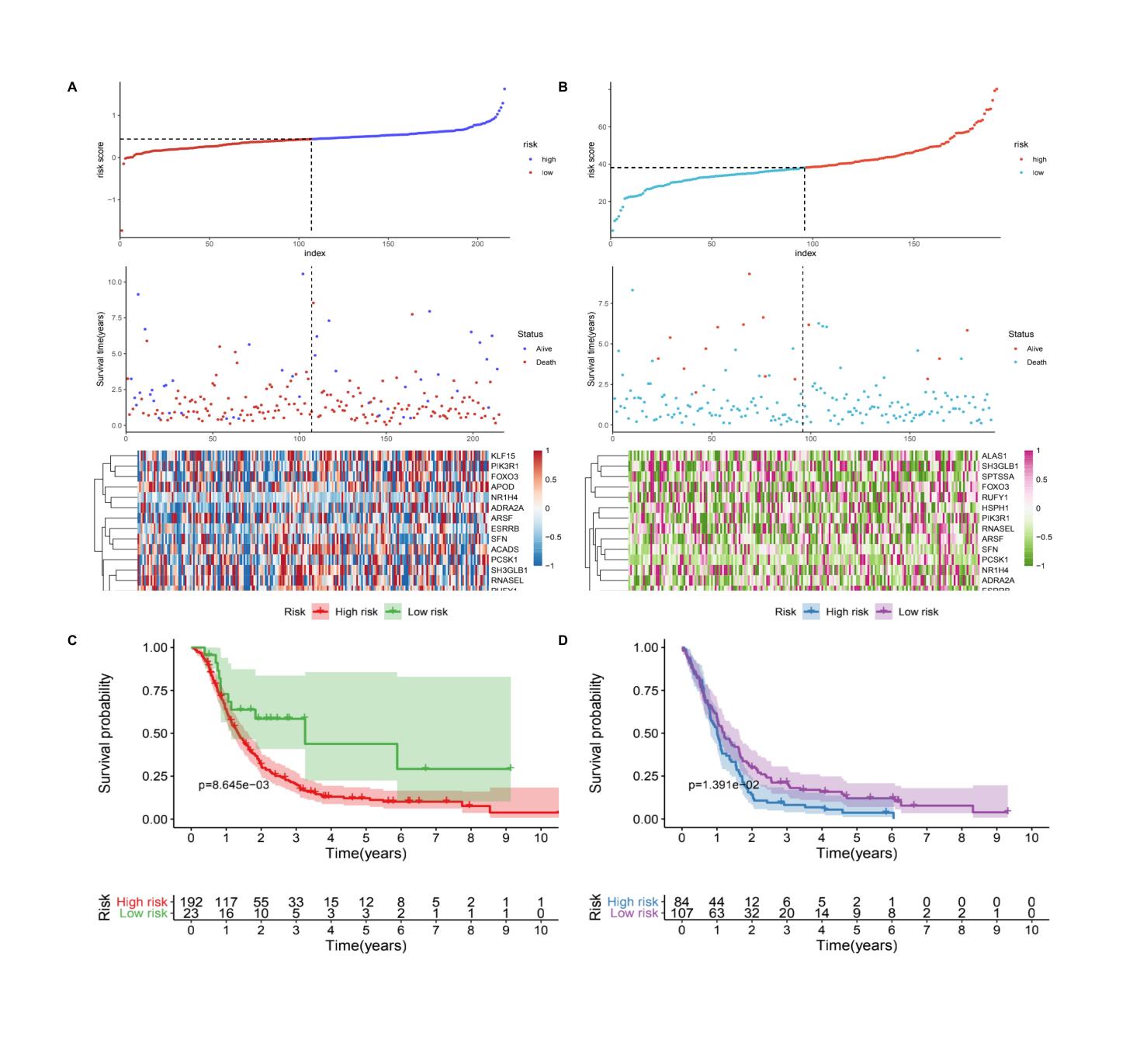


**Supplementary Figure S6.** **(A)** and **(B)** Risk plot for the GBM patients in CGGA **(A)** and GSE13041 **(B)** cohorts respectively. Each panel consists of three rows: top rows showed a risk score distribution for the high-risk score group and low-risk score group; middle rows represent the GBM patients’ distribution and survival status; the bottom rows showed that the heatmap of 17 prognostic metabolism-related genes expression. **(C)** and **(D)** Survival analysis of OS in high‐ and low‐risk groups of patients in CGGA **(C)** and GSE13041**(D)** respectively. OS, overall survival.

**
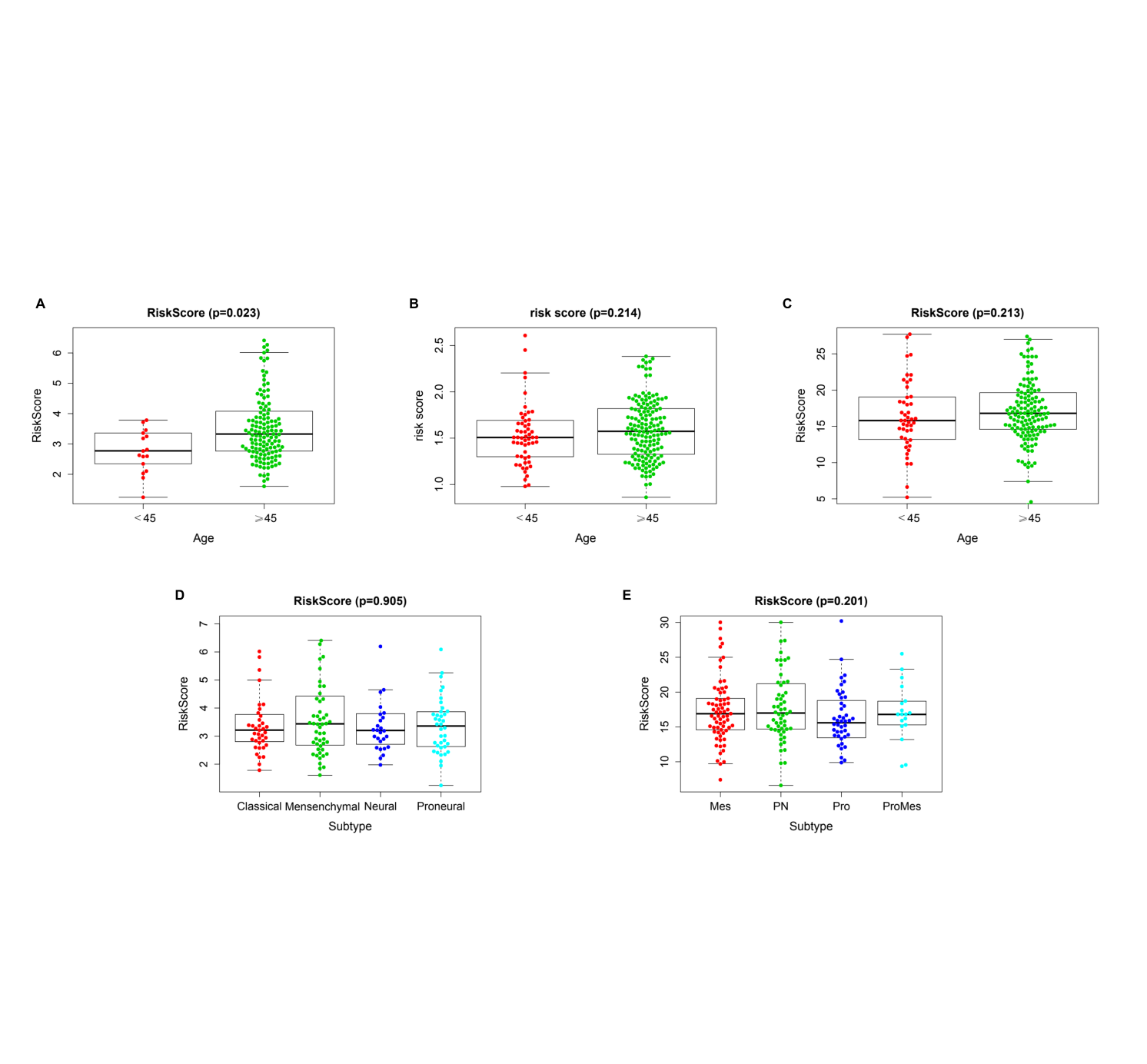
**

**Supplementary Figure S7.** Association between the metabolism‐related gene panel and age, molecular subtype. **(A)**‐**(C)** Distribution of the risk score in stratified patients by age in TCGA, CGGA and GSE13041 cohorts. **(D)**‐**(E)** Distribution of the risk score in stratified patients by molecular subtype in TCGA and GSE13041 cohorts. PN, ProNeural, Pro, Proliferative; Mes,Mesenchymal；ProMes, Pro and Mes.


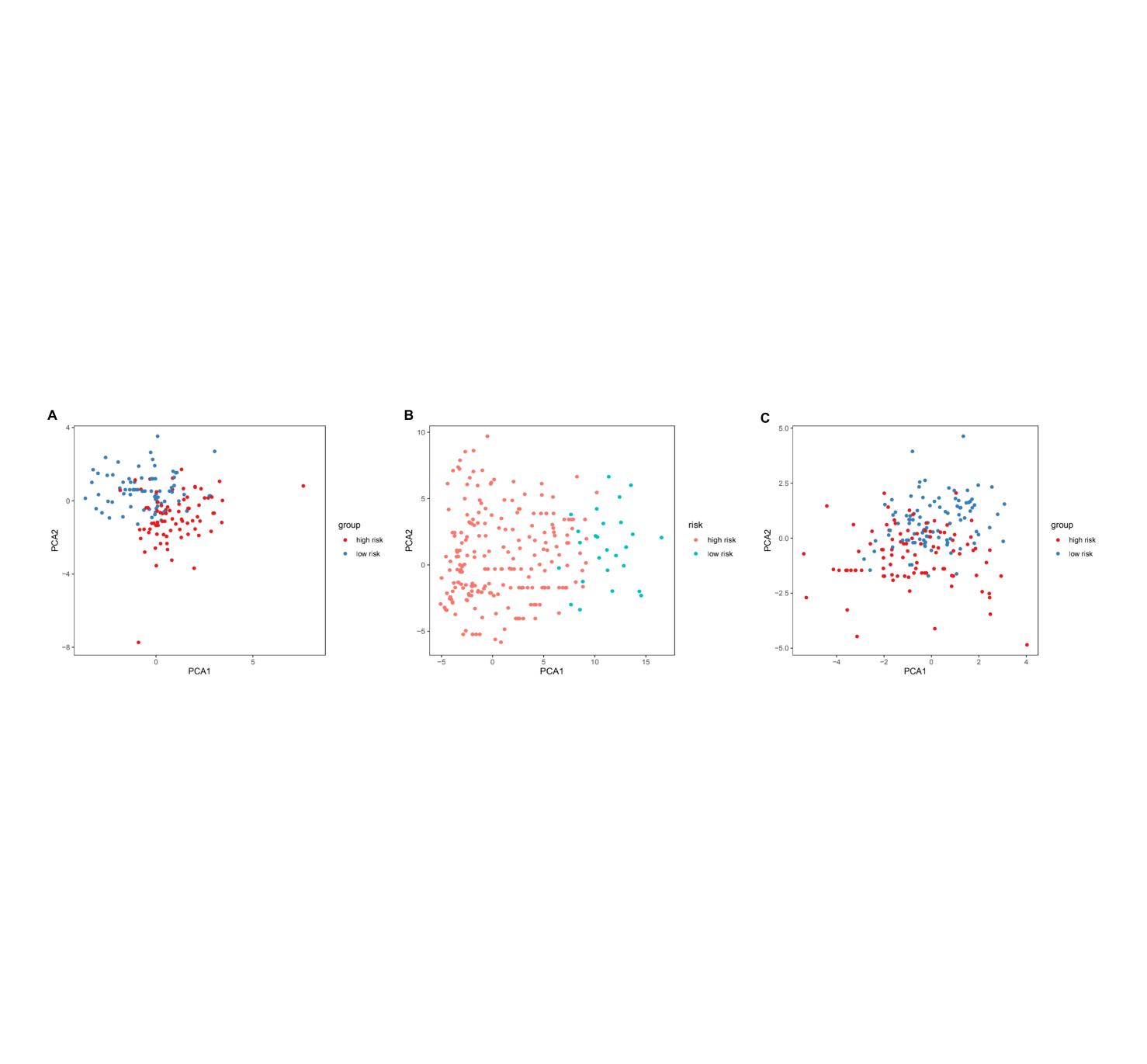


**Supplementary Figure S8.** Principal components analysis of high- and low-risk groups of patients based on top100 metabolism-related genes expression data in TCGA **(A)**, CGGA **(B)** and GSE13041 **(C)** cohorts.
